# A Label-Free Fluorescent Assay for the Rapid and Sensitive Detection of Adenosine Deaminase Activity and Inhibition

**DOI:** 10.3390/s18082441

**Published:** 2018-07-27

**Authors:** Xinxing Tang, Kefeng Wu, Han Zhao, Mingjian Chen, Changbei Ma

**Affiliations:** 1 School of Art and Design, Changsha University of Science and Technology, Changsha 410114, China; tnewstar@163.com; 2 School of Life Sciences, Central South University, Changsha 410013, China; kefeng@csu.edu.cn (K.W.); 172511001@csu.edu.cn (H.Z.); chenmingjian@csu.edu.cn (M.C.)

**Keywords:** fluorescence, label-free, adenosine deaminase, thioflavin T

## Abstract

Adenosine deaminase (ADA), able to catalyze the irreversible deamination of adenosine into inosine, can be found in almost all tissues and plays an important role in several diseases. In this work, we developed a label-free fluorescence method for the detection of adenosine deaminase activity and inhibition. In the presence of ADA, ATP has been shown to be hydrolyzed. The ATP aptamer was shown to form a G-quadruplex/thioflavin T (ThT) complex with ThT and exhibited an obvious fluorescence signal. However, the ATP aptamer could bind with ATP and exhibited a low fluorescence signal because of the absence of ADA. This assay showed high sensitivity to ADA with a detection limit of 1 U/L based on an SNR of 3 and got a good linear relationship within the range of 1–100 U/L with R^2^ = 0.9909. The LOD is lower than ADA cutoff value (4 U/L) in the clinical requirement and more sensitive than most of the reported methods. This technique exhibited high selectivity for ADA against hoGG I, UDG, RNase H and λexo. Moreover, this strategy was successfully applied for assaying the inhibition of ADA using erythro-9-(2-hydroxy-3-nonyl) adenine (EHNA) and, as such, demonstrated great potential for the future use in the diagnosis of ADA-relevant diseases, particularly in advanced drug development.

## 1. Introduction

Adenosine deaminase (ADA), a key hydrolytic enzyme in the purine metabolism, can catalyze the irreversible deamination of adenosine (deoxyadenosine) into inosine (deoxyinosine) via removal of an amino group [1,2,3]. ADA can be found in various mammals, including all human tissues, and plays a critical role in various diseases [4,5,6,7]. Interestingly, both genetic ADA deficiency and ADA overexpression may cause diseases. Generally appreciated is the notion that inherited genetic ADA deficiency represents the main cause of severe combined immunodeficiency disease (SCID), accounting for about 15% of all SCID cases [8,9,10,11]. Conversely, overexpression of ADA may be closely related to hemolytic anemia [12], liver cancer, breast cancer, etc. [13].

Given the significant role the enzyme plays in pathology, research studies on ADA have attracted significant interest. Various techniques have been used to study this enzyme type, including measuring the ammonia amount produced [14], high-performance liquid chromatography (HPLC) [15], colorimetric assay [16] and electrochemical aptasensors [17]. Unfortunately, several limitations such as generally labor-intensive processes, complex sample preparations and low selectivity impede the overall applicability of these methods. Recently, various articles highlighting studies on ADA have been reported. For example, Xu et al. developed a novel method for ATP and ADA detection based on an aptamer DNA-templated fluorescence silver nanocluster [18]. Meanwhile, Cheng et al. explored a gold nanoparticle-based label-free colorimetric aptasensor for adenosine deaminase detection and inhibition assay [19]. Feng et al. reported a fluorescence sensor for adenosine deaminase based on an adenosine-induced self-assembly of aptamer structures [20]. All of these novel methods have been shown to be effective to assay ADA. However, these methods also exhibited various disadvantages, including a time-consuming and complicated synthesis process of AgNCs or AuNCs, expensive fluorescence labeling and low sensitivity. To overcome these shortcomings, a variety of strategies have been developed, particularly involving the introduction of aptamers.

Aptamers, i.e., DNA/RNA oligonucleotides, are derived from a random sequence nucleic acid library through an in vitro selection process and are generally referred to as a systematic evolution of ligands by exponential enrichment (SELEX) [21,22,23,24]. Aptamers can be selected for a broad range of targets, from small molecules to whole cells with desirable selectivity, specificity, and affinity [25,26,27]. In addition, the synthesis, maintenance, and delivery of aptamers are relatively easy [28,29]. Hence, numerous aptamer-based sensors have been reported in the literature for the detection of a variety of target analytes [30,31,32,33]. Thioflavin T (ThT), a widely used water soluble fluorogenic dye, has been demonstrated to effectively bind to G-quadruplexes, resulting in an enhanced fluorescence signal. Because of the convenience and high sensitivity of this dye, many G-quadruplex/ThT fluorescent sensors have been proposed in the literature [34,35,36,37]. Herein, attempting to integrate the advantages of an aptamer and ThT, we propose a novel and label-free fluorescent aptasensor for the detection of adenosine deaminase activity and inhibition. Compared to currently reported methods [38,39,40], our assay provided high sensitivity and low cost.

## 2. Experimental

### 2.1. Materials and Methods

Uracil DNA glycosylase (UDG), λ exonuclease (λ_exo_) and hoGG I were obtained from New England Biolabs (Beverly, MA, USA). Ribonuclease H (RNase H) was obtained from Takara Biotechnology Co., Ltd. (DaLian, China). ATP aptamer probe (ABA) 5′-ACC TGG GGG AGT ATT GCG GAG GAA GGT-3′ was synthesized and HPLC-purified by Sangon Biotechnology Co., Ltd. (Shanghai, China). Adenosine deaminase (ADA), erythro-9-(2-hydroxy-3-nonyl) adenine (EHNA) and Thioflavin T (ThT) were purchased from Sigma-Aldrich (St. Louis, MO, USA). Ultrapure water (18.2 MΩ·cm) used in the experiments was obtained from a Milli-Q water purification system (Millipore Corp, Bedford, MA, USA). Fluorescence spectra were obtained using a Hitachi F-2700 fluorescence spectrophotometer (Hitachi Ltd., Tokyo, Japan). The samples were placed in quartz cuvettes and excited at a wavelength of 425 nm. All emission spectra were collected at wavelengths ranging from 450 to 600 nm at room temperature.

### 2.2. Fluorescent Detection of ADA

In the ADA assay, MgCl_2_ (5 mM), ATP (0.2 mM) and varying concentrations of ADA were placed in a 100 mL reaction solution (10 mM Tris–HCl, pH = 7.5). The resulting mixtures were incubated at room temperature for 20 min. Then, the ABA probe (300 nM) was added to the reaction mixtures and the samples were stored at room temperature for 20 min. Finally, ThT fluorescence dye (8 µM) was added to the above solution. After 5 min, the fluorescence intensity of the mixtures was measured using a fluorescence spectrophotometer.

### 2.3. Selectivity Assay

In order to examine the specificity of this experiment for ADA, we selected several universal enzymes such as hoGG I, UDG, RNase H and λ_exo_ as controls. In the specific experiment, MgCl_2_ (5 mM), ATP (0.2 mM) and other enzymes (1 U/mL) were incubated at room temperature for 20 min. Then, the ABA probe (300 nM) was added to the reaction solution and the resulting mixture was stored at room temperature for 20 min. ThT (8 μM) was added to the final solution and the resulting mixtures were stored at room temperature for 5 min before analysis.

### 2.4. Inhibition of ADA Activity

To demonstrate the feasibility of this method for screening ADA inhibitors, EHNA, a well-known ADA inhibitor, was selected as a model inhibitor for this study. First, MgCl_2_ (5 mM), ADA (1 U/mL) and different concentrations of EHNA were incubated at room temperature for 20 min. Subsequently, ATP (0.2 mM) was added and the reaction mixtures were stored for 20 min at room temperature. Then, the ABA probe (300 nM) was added and the resulting mixtures were kept in room temperature for 20 min. Finally, ThT (8 µM) was added and the corresponding fluorescence was measured after 5 min.

## 3. Results and Discussion

### 3.1. Principle of ADA Assay

**Figure 1:**
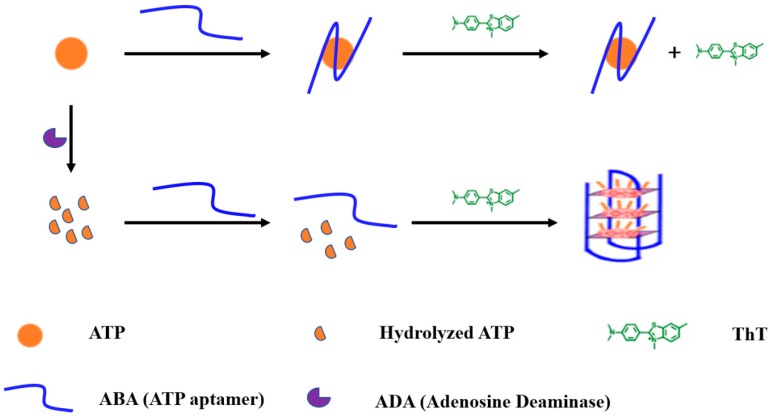
Schematic illustration of the fluorescence sensor for the detection of ADA. In the absence of ADA, the ATP aptamer could bind with ATP and showed a low fluorescence signal. With the addition of ADA, ATP was hydrolyzed. Hence, ATP aptamer could form a G-quadruplex/thioflavin T (ThT) complex with ThT and exhibited an obvious fluorescence signal.

In this assay, a label-free fluorescent aptasensor for rapid and sensitive detection of adenosine deaminase activity and inhibition is proposed. The general principle is illustrated in Figure 1. ATP aptamer (ABA) represents a special oligonucleotide containing a G-rich sequence that can specifically bind to ATP. Under certain conditions, the ABA probe may fold into a G-quadruplex and then form a G-quadruplex/ThT complex with ThT, eventually generating an obvious fluorescence signal. In the absence of ADA, the ABA probe binds to ATP. Therefore, the G-rich sequence of the ABA probe may not be able to fold into a G-quadruplex, resulting in low fluorescence. In the presence of ADA, the conversion of adenosine to inosine is catalyzed through the removal of an amino group. In this case, ATP is hydrolyzed and cannot bind to the ABA probe. However, the ABA probe may form a G-quadruplex/ThT complex with ThT, ultimately resulting in strong fluorescence. By comparing the fluorescence changes, we could quantitatively analyze the ADA activity.

### 3.2. Strategy Feasibility

**Figure 2:**
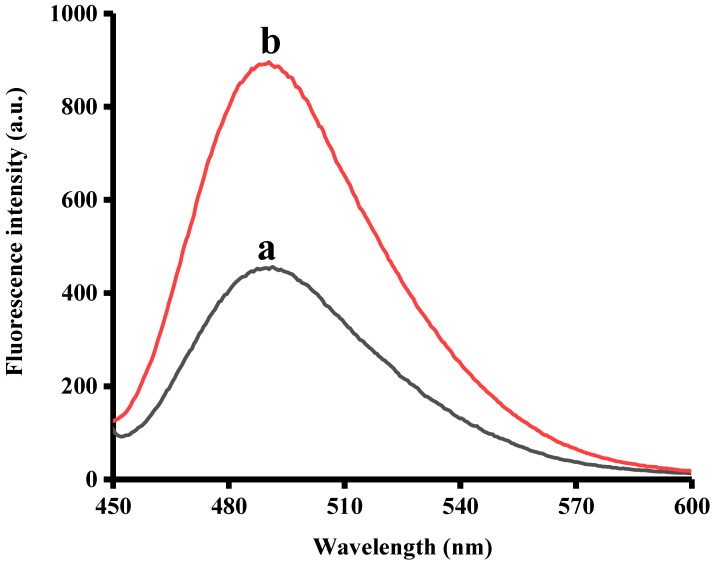
Fluorescence emission spectra in the absence (a) and presence (b) of ADA. The selected ADA concentration was 2000 U/L.

To verify the feasibility of this sensing platform, two samples were prepared. In sample A, no ADA was present. For comparison, sample B containing ADA was prepared. First, 5 mM MgCl_2_, 2 U/mL ADA (sample B) or no ADA (sample A) were added in reaction buffer and incubated at room temperature for 20 min. Subsequently, 0.2 mM ATP was added and stored for 20 min at room temperature. Then, 300 nM ABA probe was added and the resulting mixtures were kept in room temperature for 20 min. Finally, 8 µM ThT was added and fluorescence emission spectra was measured after 5 min. As demonstrated in Figure 2, the fluorescence emission intensity of sample A (curve a) was quite low. This result was indeed expected due to the absence of ADA in sample A. As described above, the ABA probe has to specifically bind to ATP, leading to the G-rich sequence of the ABA probe to fold into a G-quadruplex. Obviously, the amount of G-quadruplex/ThT complex formed in sample A was negligible and, as such, the fluorescence signal was insignificant. However, sample B (curve b) exhibited a high fluorescence signal intensity. This finding was mainly due to the presence of ADA, whereas ATP was hydrolyzed and could not bind to the ABA probe. In contrast to sample A, the ABA probe in sample B could form a G-quadruplex/ThT complex with ThT and express remarkable fluorescence.

### 3.3. Optimization of Assay Conditions

**Figure 3:**
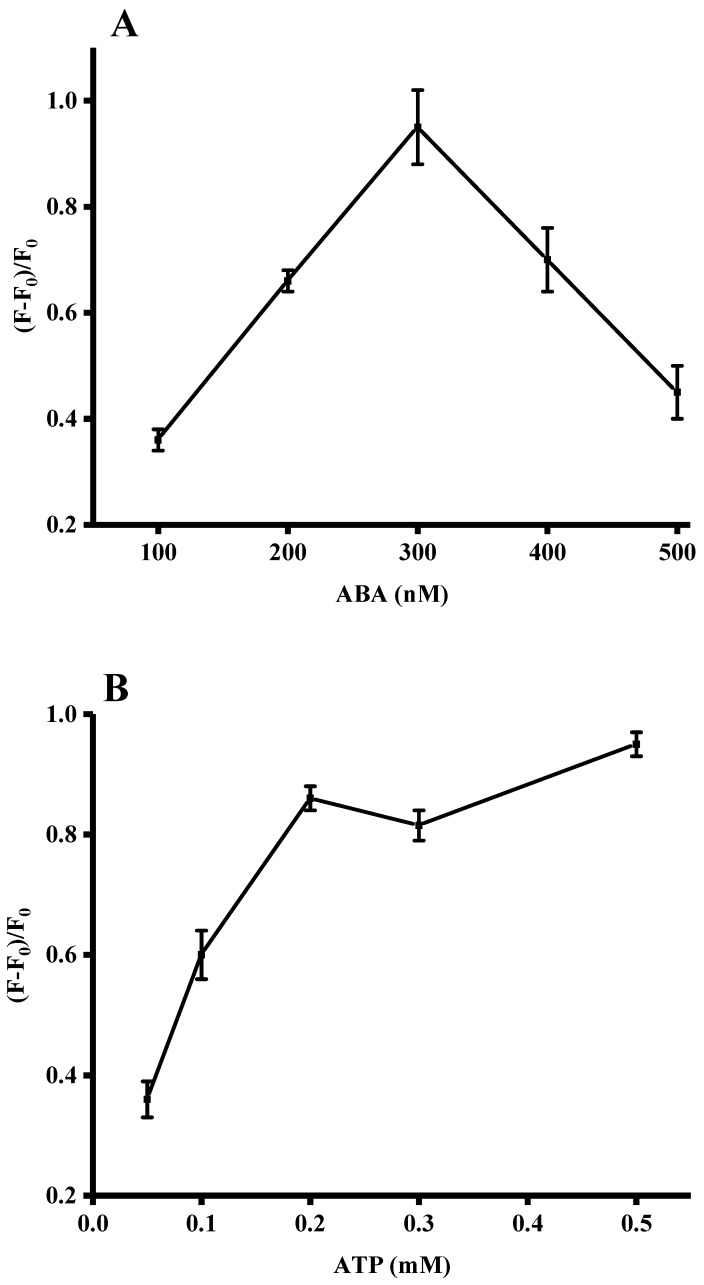

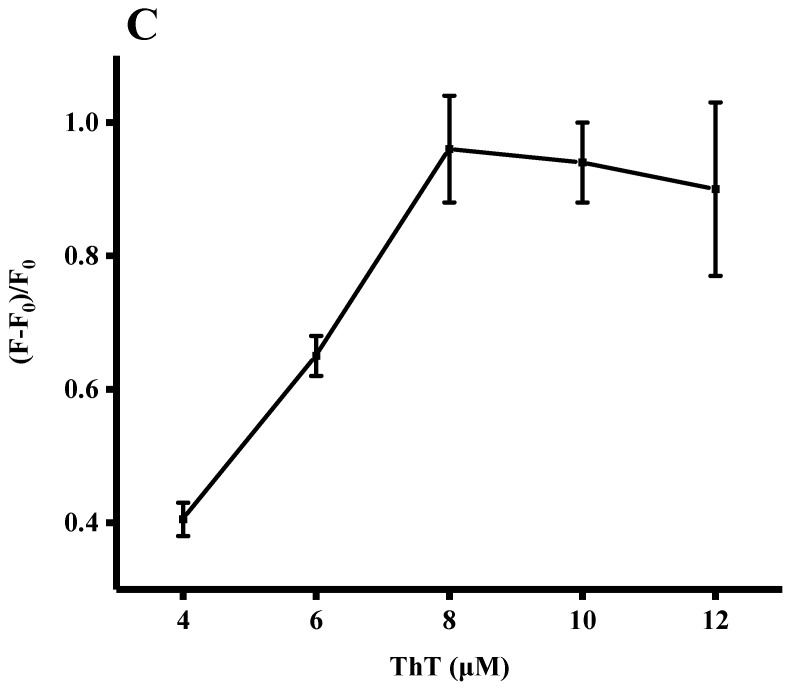
Optimization of experimental conditions for ADA detection. (**A**) Concentration of ABA probe (100, 200, 300, 400 and 500 nM), ATP (0.2 mM), and ThT (8 μM); (**B**) concentration of ATP (0.05, 0.1, 0.2, 0.3 and 0.5 mM), ABA (300 nM), and ThT (8 μM); (**C**) concentration of ThT (4, 6, 8, 10 and 12 μM), ABA (300 nM), and ATP (0.2 mM). Error bars were estimated from three replicate measurements.

In order to achieve good performance of this sensing system for ADA detection, a series of experimental conditions were optimized. Obviously, the concentration of the ABA probe had an important influence on the formation of the amount of G-quadruplex/ThT complexes. As shown in Figure 3A, the fluorescence intensity increased gradually upon addition of different ABA probe concentrations (100, 200, 300, 400 and 500 nM) to the sensing system. When the concentration of the ABA probe was higher than 300 nM, the fluorescence signal intensity declined. Thus, 300 mM was used as the optimal concentration of the ABA probe in all following experiments. Since the ABA probe could specifically bind to ATP and since the probe was structurally related to the structure of ADA, the presence of the latter may significantly affect this sensing platform. Therefore, the concentration of ATP was optimized by carrying out a series of assays at different ATP concentrations (0.05, 0.1, 0.2, 0.3 and 0.5 mM). As shown in Figure 3B, when the concentration of ATP reached a concentration of 0.2 mM, the fluorescence signal increase tended to be stable. Thus, an ATP concentration of 0.2 mM was selected in subsequent experiments. As shown in Figure 3C, ThT concentrations of 4, 6, 8, 10 and 12 μM were selected. The ratio of fluorescence intensity increase reached a plateau at a ThT concentration of 8 μM. Thus, a ThT concentration of 8 μM was used throughout the following experiments. The optimal assay conditions were: ABA (300 nM), ATP (0.2 mM), and ThT (8 µM).

### 3.4. Quantitative Detection of ADA

**Figure 4:**
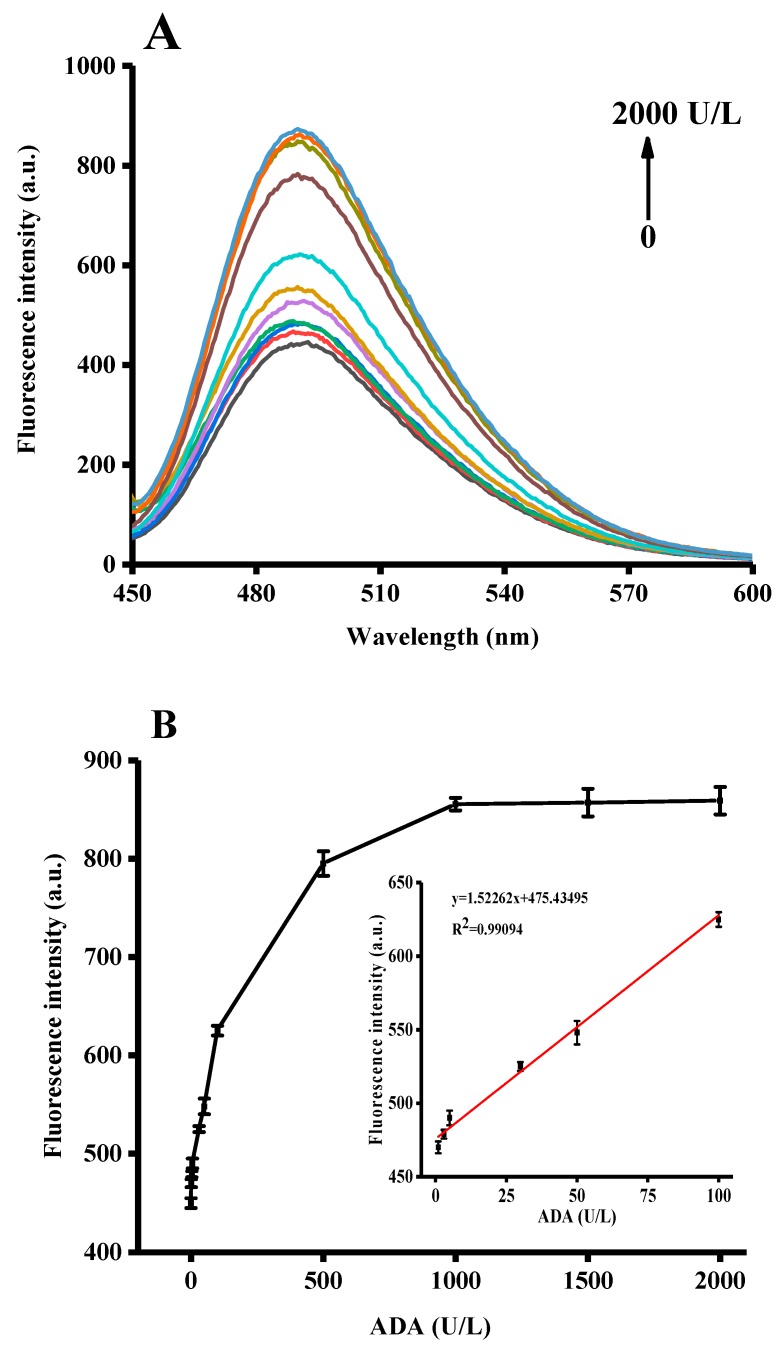
(**A**) Fluorescence emission spectra of aptasensor complex in the presence of increasing ADA concentrations (0, 1, 3, 5, 30, 50, 100, 500, 1000, 1500, 2000 U/L); (**B**) Change of fluorescence intensity versus ADA concentration. The inset shows the linear correlation at low ADA concentrations. Error bars were estimated from three replicate measurements.

Under optimal experimental conditions, we investigated the fluorescence responses of the as-proposed assay to different ADA concentrations. As shown in Figure 4A, the fluorescence intensity was enhanced as the concentration of ADA increased from 0 to 2000 U/L, suggesting that the formation of the G-quadruplex/ThT complex was highly dependent on the concentration of ADA. The calibration curve for the detection of ADA activity is shown in Figure 4B. The detection limit was estimated to be 1 U/L based on an SNR of 3, comparable to or better than some other commonly used techniques (cf. Table 1). As illustrated in the inset of Figure 4B, the fluorescence intensity exhibited a linear correlation to the activity of ADA ranging from 1 to 100 U/L, with an R^2^ of 0.9909.

### 3.5. Selectivity of ADA Assay

**Figure 5:**
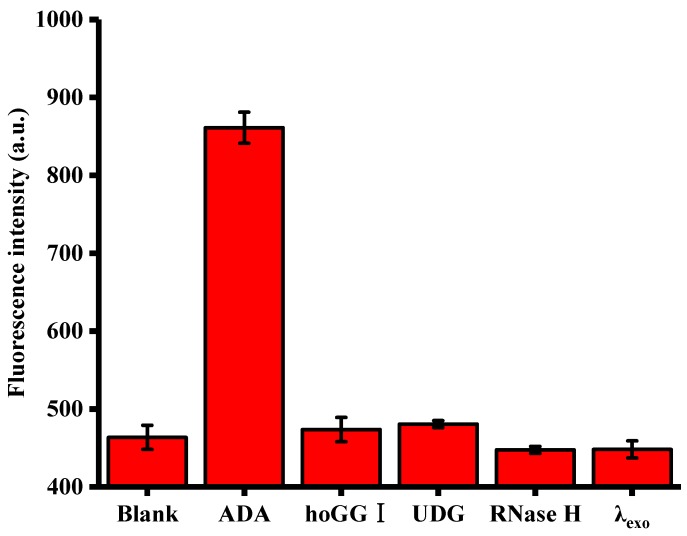
Relative fluorescence intensity of the reaction systems upon addition of ADA, hoGG I, UDG, RNase H and λexo. Error bars were estimated from three replicate measurements.

The specificity of the method was tested using hoGG I, UDG, RNase H and λexo as controls. The experiments were performed using a concentration of 1000 U/L for all enzyme types in this reported assay. It is clear from inspection of Figure 5 that under identical conditions, a remarkable fluorescence signal was observed in response to ADA, while no significant fluorescence changes could be detected in the presence of the reaction buffer, hoGG I, UDG, RNase H and λexo. Taken in concert, these results demonstrate the excellent specificity of the proposed assay towards ADA.

### 3.6. Detection of the ADA Inhibitor

**Figure 6:**
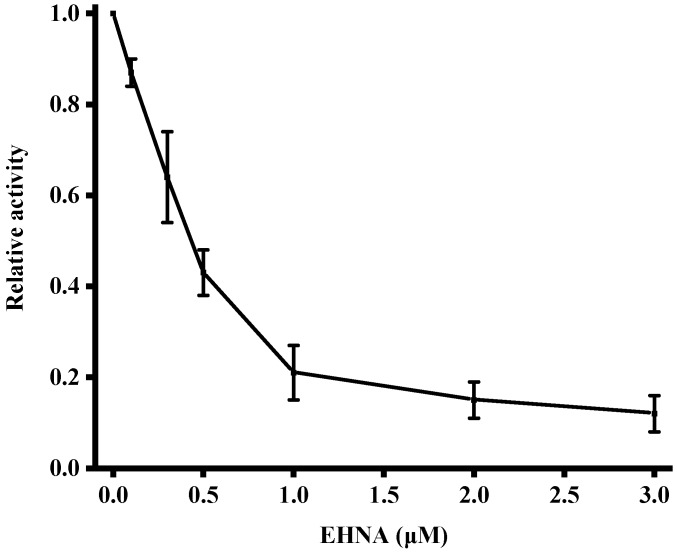
Variance of relative activity of ADA in response to different concentrations of EHNA. Error bars were estimated from three replicate measurements.

Because of the importance of excess ADA in pathology, inhibitors of ADA may have potential clinical applications. Through the inhibitor experiment, it can also provide a research basis for anti-ADA drug. We have conducted the studies on the inhibition of enzymatic activity by incubating ADA with different concentrations of EHNA, a known inhibitor of ADA. The concentration of ADA was fixed at 1000 U/L. As expected, the relative activity of ADA decreased upon increasing the amount of EHNA. As shown in Figure 6, this result confirmed that the developed strategy could be used for the screening of ADA inhibitors.

## 4. Conclusions

In summary, we developed a simple, sensitive and selective assay of ADA activity and its inhibitor using an ATP aptamer. This label-free method could quantify ADA activity as low as 1 U/L, more sensitive than most of the existing approaches currently available. Simultaneously, this proposed assay featured a detection range that was shown to be linear from 1 U/L to 100 U/L, with a correlation coefficient of 0.9909. In addition, the method could be successfully applied for the evaluation of the inhibition effect of EHNA on ADA activity. The results also revealed that this strategy may be potentially applied in ADA-related clinical diagnosis and functional research.

## Figures and Tables

**Table 1:** Comparison with the currently reported methods for ADA determination.

Biosensing Principle	LOD (U/L)	Linear Range (U/L)	Reference
GO based fluorescent aptasensor	12.9	0–240	[13]
Electrochemical aptasensor	200	0–10,000	[17]
Silver nanocluster	5	--	[18]
Gold nanoparticle	1.5	4.2–21	[19]
Mass spectrometry	0.5	--	[38]
Label-free aptasensor	2	0–20	[39]
Electrochemical aptasensor	1	0–100	[40]
ThT	1	1–100	This work
